# Quantitative Proteomics Explore the Potential Targets and Action Mechanisms of Hydroxychloroquine

**DOI:** 10.3390/molecules27165175

**Published:** 2022-08-14

**Authors:** Jingxiang Zhao, Zhiqiang Zhao, Wanting Hou, Yue Jiang, Guobin Liu, Xuelian Ren, Kun Liu, Hong Liu, Kaixian Chen, He Huang

**Affiliations:** 1 School of Pharmacy, China Pharmaceutical University, Nanjing 211198, China; 2 Shanghai Institute of Materia Medica, Chinese Academy of Sciences, Shanghai 201203, China; 3 School of Life Science and Technology, ShanghaiTech University, Shanghai 201210, China; 4 School of Mechanical Engineering and Automation, Northeastern University, Shenyang 110819, China; 5 University of Chinese Academy of Sciences, Beijing 100049, China

**Keywords:** hydroxychloroquine, quantitative proteomics, thermal proteome profiling

## Abstract

Hydroxychloroquine (HCQ) is an autophagy inhibitor that has been used for the treatment of many diseases, such as malaria, rheumatoid arthritis, systemic lupus erythematosus, and cancer. Despite the therapeutic advances in these diseases, the underlying mechanisms have not been well determined and hinder the rational use of this drug in the future. Here, we explored the possible mechanisms and identified the potential binding targets of HCQ by performing quantitative proteomics and thermal proteome profiling on MIA PaCa-2 cells. This study revealed that HCQ may exert its functions by targeting some autophagy-related proteins such as ribosyldihydronicotinamide dehydrogenase (NQO2) and transport protein Sec23A (SEC23A), or regulating the expression of galectin-8 (LGALS8), mitogen-activated protein kinase 8 (MAPK8), and so on. Furthermore, HCQ may prevent the progression of pancreatic cancer by regulating the expression of nesprin-2 (SYNE2), protein-S-isoprenylcysteine O-methyltransferase (ICMT), and cotranscriptional regulator FAM172A (FAM172A). Together, these findings not only identified potential binding targets for HCQ but also revealed the non-canonical mechanisms of HCQ that may contribute to pancreatic cancer treatment.

## 1. Introduction

**Figure 1:**
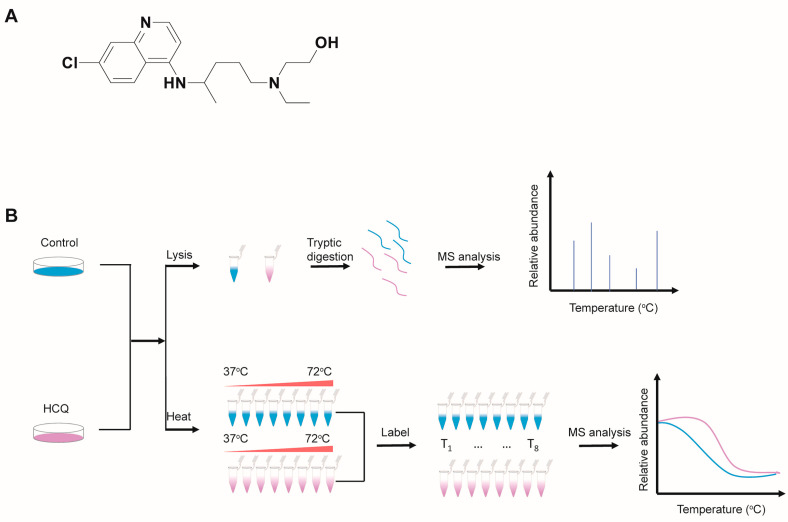
Workflow of the quantitative proteomics and the thermal proteome profiling studies. (**A**) The chemical structure of HCQ. (**B**) Overall workflows of the quantitative proteomics and the thermal proteome profiling. MIA PaCa-2 cells were treated with or without HCQ, respectively. For the quantitative proteomics study, cell lysates were digested with trypsin and analyzed by LC-MS/MS; for the thermal proteome profiling, cells were equally divided into eight aliquots, heated from 37 °C to 72 °C, and the samples were analyzed by LC-MS/MS after TMT labeling.

Hydroxychloroquine (HCQ), a derivative of chloroquine (Figure 1A), was primarily used to treat malaria, especially in areas where malaria remains sensitive to chloroquine [1]. Over the past half-century, the benefit of HCQ has gradually expanded to many other diseases and HCQ has become one of the most commonly prescribed medications [2]. Currently, HCQ is widely used for the treatment of rheumatoid arthritis [3], systemic lupus erythematosus [3], and antiphospholipid antibody syndrome [4]. Because HCQ can accumulate in lysosomes and other acidic compartments to make them alkalize [5], it shows antibacterial [6], antifungal [7], and antiviral [8] activity, and is used to treat some infectious diseases. For example, Whipple’s disease, a chronic bacterial infective disease in the intestinal mucosa caused by *Tropheryma whippeli*, can be treated with a combination of doxycycline and HCQ [9]. Another important effect of HCQ worth highlighting is its antitumor activity [10]. It shows an additive effect in combination with conventional antitumor therapies, chemotherapy, and radiotherapy [10]. Many clinical trials [2] are ongoing to evaluate the effects of HCQ on several types of cancer, such as pancreatic cancer [11], non-small cell lung cancer [12], and multiple myeloma [13]. 

Traditionally, HCQ has been thought to act by inhibiting autophagy, a cell survival pathway enabling cells to recoup metabolic fuel by degrading damaged proteins and organelles [14,15]. For example, it has been reported that increased autophagy is a metabolic requirement for pancreatic cancer [16] and autophagy can degrade MHC-I to promote immune evasion of pancreatic cancer [17]. Despite the progress, the target proteins of HCQ to inhibit autophagy and the precise mechanisms by which HCQ enhances tumor cell death remain largely unknown. Therefore, revealing the mechanisms of action and identifying the potential binding targets of HCQ is essential to improving clinical outcomes.

To achieve the above goal, we carried out quantitative proteomics to explore the possible mechanisms of action by revealing the dynamically expressed proteins induced by HCQ and performed a thermal proteome profiling study to identify the potential target proteins of HCQ. Interestingly, several autophagy-related proteins, such as NQO2, SEC23A, pericentriolar material 1 protein (PCM1), and ADP-ribosylation factor GTPase-activating protein 1 (ARFGAP1), were identified as potential binding targets of HCQ. In addition, quantitative proteomics results showed that HCQ could regulate the expression of some proteins that are involved in autophagy, including next to BRCA1 gene 1 protein (NBR1), gamma-aminobutyric acid receptor-associated protein-like 2 (GABARAPL2), LGALS8, MAPK8, and tumor necrosis factor alpha-induced protein 8-like protein 1 (TNFAIP8L1). Cellular pathway analysis of the dynamic proteome induced by HCQ showed that the upregulated and downregulated proteins are mainly enriched in cellular metabolism and rRNA biology-related processes, respectively. Moreover, the expression of some proteins that are associated with the progression of pancreatic cancer, such as SYNE2, ICMT, and FAM172A, is regulated by HCQ. Therefore, the results from our study point toward potential binding targets of HCQ and possible mechanisms responsible for the treatment of diverse diseases such as pancreatic cancer. 

## 2. Results

### 2.1. Overview of the Quantitative Proteomics and Thermal Proteome Profiling Studies

To identify the protein expression dynamics induced by HCQ, we carried out a proteomics study using MIA PaCa-2 cells treated with or without HCQ, respectively. After treatment, the samples were analyzed by LC-MS/MS (Figure 1B). The experiments were performed in triplicate and the LFQ algorithm embedded in MaxQuant software was used for the quantitative proteomics analysis. 

In the thermal proteome profiling study, MIA PaCa-2 cells were treated with or without HCQ for 1.5 h, respectively. Then, each group was divided into eight aliquots for gradient heating. The soluble proteins in the supernatant of each aliquot were labeled with TMT reagents. Subsequently, these samples were analyzed by LC-MS/MS and the proteins were identified and quantified by MaxQuant (Figure 1B). Because HCQ may change the stability of its binding proteins, we can identify the potential binding partners by determining the melting curve shift through thermal proteome profiling [18]. To improve the reliability of the data, we performed two replicate experiments for each condition.

For the data analysis, the false discovery rate was set as 1% at peptide and protein levels in both the quantitative proteomics and the thermal proteome profiling studies. 

### 2.2. Dynamics of the Proteome Induced by HCQ

**Figure 2:**
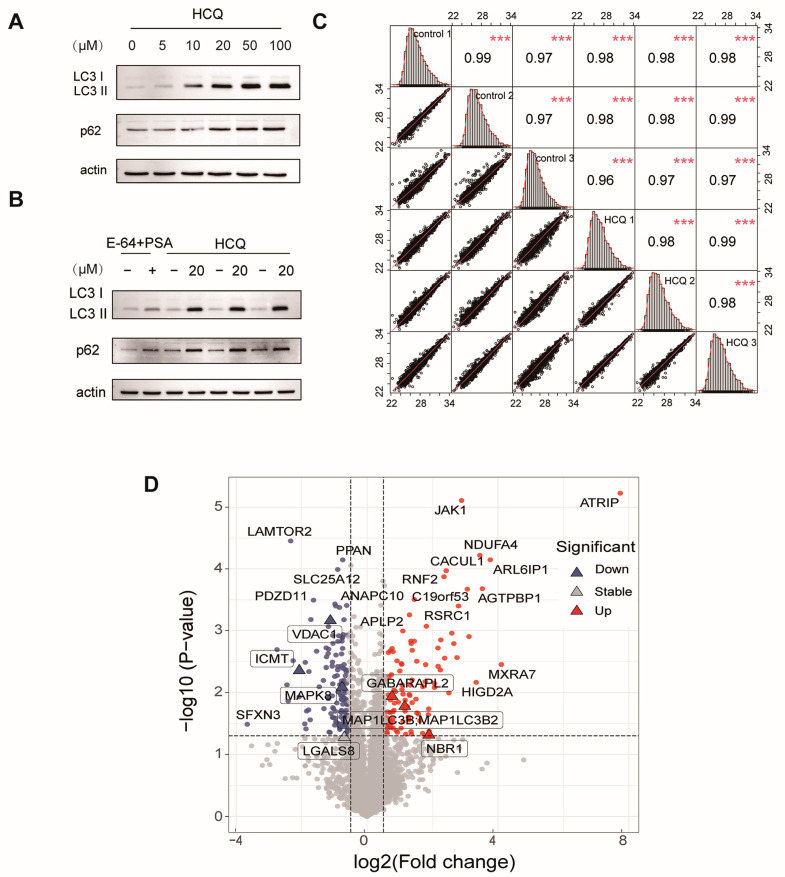
The influence of HCQ on the proteome. (**A**) HCQ inhibited autophagy dose-dependently. (**B**) Autophagy was well blocked in each replicate for the quantitative proteomics study. (**C**) The correlation between the three replicates (***, *p* < 0.001). (**D**) The volcano map showed the protein dynamics in response to HCQ treatment. Gray: unchanged proteins; red: upregulated proteins; blue: downregulated proteins.

To determine the appropriate dosing concentration, we treated MIA PaCa-2 cells with HCQ ranging from 0 μM to 100 μM. The result showed that HCQ inhibited autophagy in a dose-dependent manner (Figure 2A). The LC3-II formation and the accumulation of p62 were obviously increased at the concentration of 10 μM but slightly increased at the concentration above 20 μM. To further evaluate the autophagy inhibition of HCQ, we treated MIA PaCa-2 cells with a combination of pepstatin A (PSA) and E-64 (10 μM for each) as a positive control. The results showed that HCQ notably inhibited the autophagic flux at 20 μM, even overmatching the positive control (Figure 2B). Therefore, we treated MIA PaCa-2 cells with 20 μM of HCQ for the quantitative proteomics study. The experiments were performed in triplicate, and the Western blot analysis confirmed that the autophagy was well blocked in each replicate (Figure 2B). To confirm the reproducibility of the quantitative proteome, we calculated the correlation of data in the HCQ-treated or control group in three replicates, respectively. The results showed that the correlation between the three replicates fitted well, with coefficients of about 1 (Figure 2C). 

**Figure 3:**
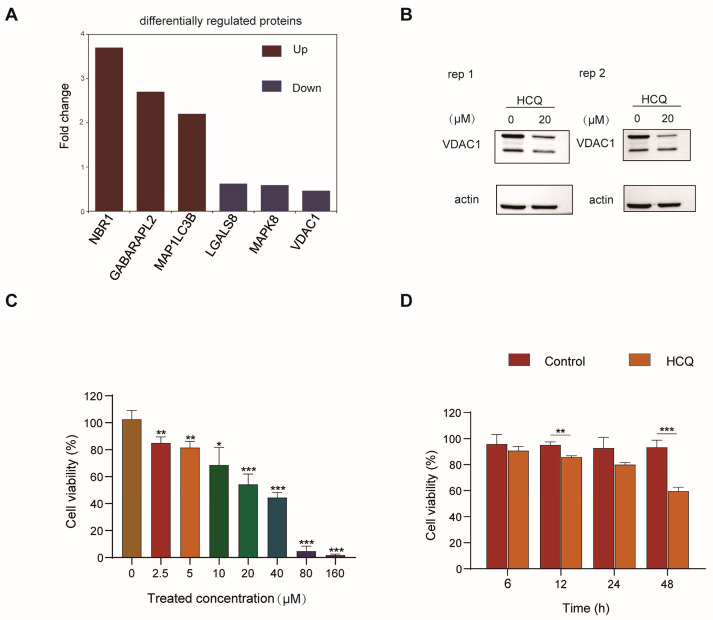
The validation of dynamic proteins induced by HCQ. (**A**) The representative regulated proteins induced by HCQ. Red, upregulated proteins; blue, downregulated proteins. (**B**) The Western blot validated the downregulation of VDAC1 induced by HCQ. (**C**) Cell viability of MIA PaCa-2 cells treated with different concentrations of HCQ for 48 h by Cell Counting Kit 8 (CCK-8) assay (*, *p* < 0.05; **, *p* < 0.01; ***, *p* < 0.001). (**D**) Cell viability of MIA PaCa-2 cells treated with 20 μM HCQ for 0, 6, 12, 24, and 48 h by CCK-8 assay (*, *p* < 0.05; **, *p* < 0.01; ***, *p* < 0.001).

In total, the proteomics study quantified 5581 proteins (Table S1 in Supplementary Materials), in which 134 proteins were downregulated (log_2_(HCQ/Control) < −0.585 and *p* < 0.05) and 102 proteins were up-regulated (log_2_(HCQ/Control) > 0.585 and *p* < 0.05) (Figure 2D). As expected, the expression of microtubule-associated proteins 1A/1B light chain 3B (MAP1LC3B) increased by 1.2-fold after HCQ treatment, which was consistent with the immunoblotting (Figure 3A). Interestingly, some dynamic proteins were reported to be involved in autophagy. For example, NBR1, a protein that contains LC3 and ubiquitin-binding domains, is recruited to ubiquitinated protein aggregates and undergoes autophagic degradation through the LC3 interacting region and LC3 family modifiers, serving as an autophagy substrate [19]. In our results, the expression of NBR1 was upregulated by 270% in response to HCQ treatment (Figure 3A), suggesting that autophagy may be inhibited by HCQ through blocking the fusion of phagosomes and lysosomes. GABARAPL2, an ATG8 ortholog belonging to GATE-16/GABARAP subfamilies, plays an important role in autophagosome formation and the elongation of the membrane [20]. In our results, the level of GABARAPL2 was increased to 170% after HCQ treatment (Figure 3A), suggesting that the HCQ may inhibit the autophagosome formation and elongation of the membrane. LGALS8 is a beta-galactoside-binding lectin that plays a role in restricting the proliferation of infecting pathogens by targeting them for autophagy [21]. 

When lysosomes are damaged, LGALS8 closes to sodium-coupled neutral amino acid transporter 9 (SLC38A9) which can translocate mTOR from the lysosomal membrane to the cytoplasm, in turn inactivating mTOR and activating autophagy [22]. The abundance of LGALS8 decreased by 38% upon HCQ treatment (Figure 3A), which might lead to mTOR activation and autophagy inhibition by impeding the dissociation of mTOR from the damaged lysosome. Another protein worth mentioning is MAPK8. Under starvation conditions, MAPK8 induces phosphorylation of Bcl-2 and activates autophagy [23]. Interestingly, HCQ treatment decreased the MAPK8 expression level by 41% (Figure 3A). Given the important role of MAPK8 in the regulation of Bcl-2 phosphorylation, this result suggested that HCQ may inhibit autophagy by decreasing phosphorylation of Bcl-2 mediated by MAPK8. 

It has been reported that upregulation of voltage-dependent anion-selective channel protein 1 (VDAC1) led to cardiomyocyte autophagy through the PINK1/parkin pathway [24]. Our results showed that the expression of VDAC1 decreased by 54% after HCQ treatment (Figure 3A). To confirm the results, we performed a Western blot analysis to validate the expression dynamics of VDAC1 in MIA PaCa-2 cells in response to HCQ treatment. In consist with the mass data, the level of VDAC1 was obviously decreased after HCQ treatment, which suggested the reliability of our proteomics study (Figure 3B).

Interestingly, the quantitative proteomics study results showed that HCQ treatment decreased the expression of nesprin-2 (SYNE2) and protein-S-isoprenylcysteine O-methyltransferase (ICMT) by 81.7% and 76.3% (Table S1), respectively. SYNE2 is a nuclear outer membrane protein that plays important role in the maintenance of nucleus structural integrity. It was reported that the knockdown of SYNE2 in pancreatic ductal adenocarcinoma cells reduced their invasive activities and survival [25]. In addition, suppression of ICMT, a protein catalyzing the post-translational methylation of isoprenylated C-terminal cysteine residues, has been shown to inhibit proliferation and induce apoptosis of pancreatic cancer cells [26]. Given the important roles of the HCQ-induced proteins in cell survival, we next performed a cell viability assay to validate the anti-proliferative effect of HCQ on MIA PaCa-2 cells. As expected, the viability of the MIA PaCa-2 cells decreased in a dose-dependent and time-dependent manner after HCQ treatment (Figure 3C,D). 

### 2.3. Cellular Pathway Analysis of the Dynamic Proteins Induced by HCQ

**Figure 4:**
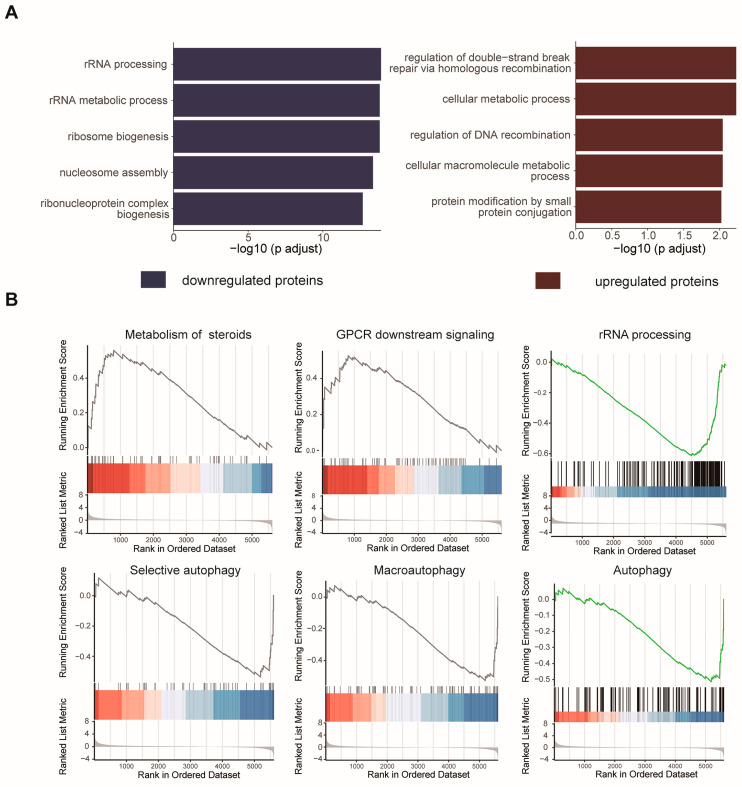
The biological functions and pathways influenced by the differentially expressed proteins. (**A**) Biological processes enriched with the dynamic proteome induced by HCQ. (**B**) Reactome pathways of the dynamic proteome induced by HCQ.

To elucidate the cellular processes affected by HCQ, we performed an enrichment analysis of the dynamic proteins induced by HCQ using Gene Ontology (GO) databases (Figure 4A) (Table S2). In the biological process category, upregulated proteins were mainly enriched in the regulation of double-strand break repair via homologous recombination (adjusted *p* = 5.9 × 10^−3^), cellular metabolic process (adjusted *p* = 5.9 × 10^−3^) and regulation of DNA recombination (adjusted *p* = 9.1 × 10^−3^), while downregulated proteins were enriched in rRNA processing (adjusted *p* = 1.28 × 10^−14^), rRNA metabolic process (adjusted *p* = 1.56 × 10^−14^), and ribosome biogenesis (adjusted *p* = 1.56 × 10^−14^) (Figure 4A). These results suggested that HCQ is highly involved in cellular metabolic processes, which is not surprising because mounting evidence has demonstrated that autophagy is closely related to metabolic diseases, such as insulin resistance, sarcopenic obesity, and type 2 diabetes mellitus. These diseases are resulting from the accumulation of lipid droplets, protein aggregates, and damaged organelles with impaired autophagy [27]. In addition, it has been reported that autophagy is highly related to RNA processes. For example, a long noncoding RNA (lncRNA) CA7-4 can regulate autophagy and apoptosis of high glucose-induced vascular endothelial cells by competing with MIR877-3P and MIR5680 [28].

Next, to investigate the possible pathways influenced by the differentially expressed proteins induced by HCQ, we performed enrichment analysis with the Reactome pathway database (Figure 4B) (Table S3). Notably, the downregulated proteins were enriched in rRNA processing (adjusted *p* = 4.7 × 10^−2^), selective autophagy (adjusted *p* = 4.38 × 10^−2^), macroautophagy (adjusted *p* = 4.38 × 10^−2^), and autophagy (adjusted *p* = 4.38 × 10^−2^) (Figure 4B), which is consistent with the phenotype and sheds some lights on the action mechanism of HCQ in autophagy inhibition. Interestingly, VDAC1 was involved in the above-enriched autophagy pathways, suggesting that HCQ may play an important role in autophagy pathways by downregulating VDAC1. The upregulated proteins are highly enriched in metabolism of steroids (adjusted *p* = 4.38 × 10^−2^) and GPCR downstream signaling (adjusted *p* = 4.76 × 10^−2^) (Figure 4B). G protein-coupled receptors (GPCRs), which can directly detect extracellular nutrients, are involved in the regulation of autophagy through multiple downstream signals, such as adenylyl cyclase (AC), ERK1/2, and mTOR [29]. Our results suggest that HCQ may inhibit autophagy by mediating the expression of proteins related to signaling cascades of GPCRs.

**Figure 5:**
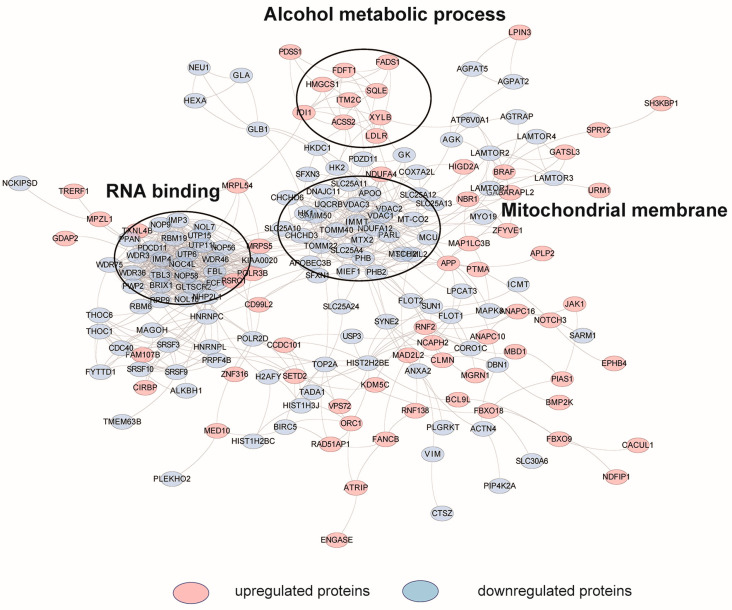
Interaction network of the differentially expressed proteins induced by HCQ, based on STRING database. The network is visualized in Cytoscape. Red color represents the upregulated proteins and blue color represents the downregulated proteins.

To further reveal the mechanism of HCQ to inhibit autophagy and the network in which the differentially expressed proteins are involved, we performed the protein-protein interactions (PPIs) analysis based on the STRING database (v11, European Molecular Biology Laboratory (EMBL), Rome, Italy, http://www.string-db.org/, accessed on 19 January 2019) and the functional enrichment of key networks of PPIs with the StringAPP (v1.7.0, National Institute of General Medical Sciences (NIGMS), Maryland, MD, USA, https://apps.cytoscape.org, accessed on 4 July 2022) embedded in Cytoscape (v.3.8.2, National Institute of General Medical Sciences (NIGMS), Maryland, MD, USA, https://cytoscape.org/, accessed on 4 July 2022) [30]. As shown in the interaction network, the upregulated proteins were related to alcohol metabolic process while the downregulated proteins were clustered in RNA binding and mitochondrial membrane (Figure 5).

### 2.4. Thermal Proteome Profiling of MIA PaCa-2 Cells Treated with HCQ

**Figure 6:**
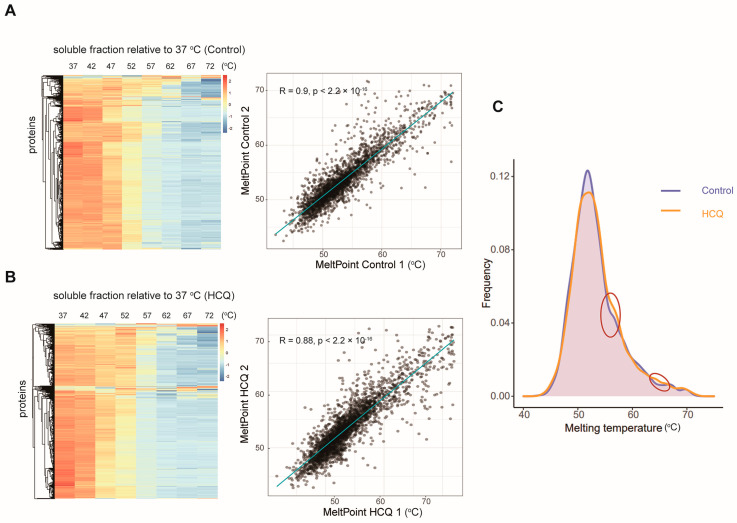
Thermal proteome analysis of the soluble proteins. (**A**) Heat map of the soluble proteins in the control group. Relative abundances of the 4144 soluble proteins at the corresponding temperature compared with the lowest temperature (37 °C) are clustered and shown. The correlation between the two replicates is 0.9. (**B**) Heat map of the soluble proteins treated with HCQ. Relative abundance of the 4327 soluble proteins at the corresponding temperature compared with the lowest temperature (37 °C) are clustered and shown. The correlation between the two replicates is 0.88. (**C**) Melting points of the total proteins in the control (purple) or HCQ-treated group (orange) are shown. The circle represents the proteins whose melting points increased after HCQ treatment.

To identify the potential binding proteins of HCQ, we performed a thermal proteome profiling study of HCQ in MIA PaCa-2 cells. In total, 4144 and 4327 soluble proteins were identified in the HCQ-treated and the control groups, respectively. With the increase in the temperature, proteins become unstable and the abundance of the soluble proteins decreased gradually (Figure 6A,B). To confirm the reproducibility of the thermal proteome profiling, we correlated melting points of the soluble proteins in the HCQ-treated or control group in two replicates, respectively. The results showed that the melting points between the two replicates fitted well, with coefficients of about 0.9 (Figure 6A,B). Next, we compared the melting point distribution of the soluble proteins in the HCQ-treated group and the control group. As shown in Figure 6C, their distributions are similar, with only a small fraction of proteins influenced by HCQ treatment, suggesting that the potential binding proteins of HCQ may locate in these areas. 

### 2.5. Identification of the Potential Binding Proteins of HCQ

**Figure 7:**
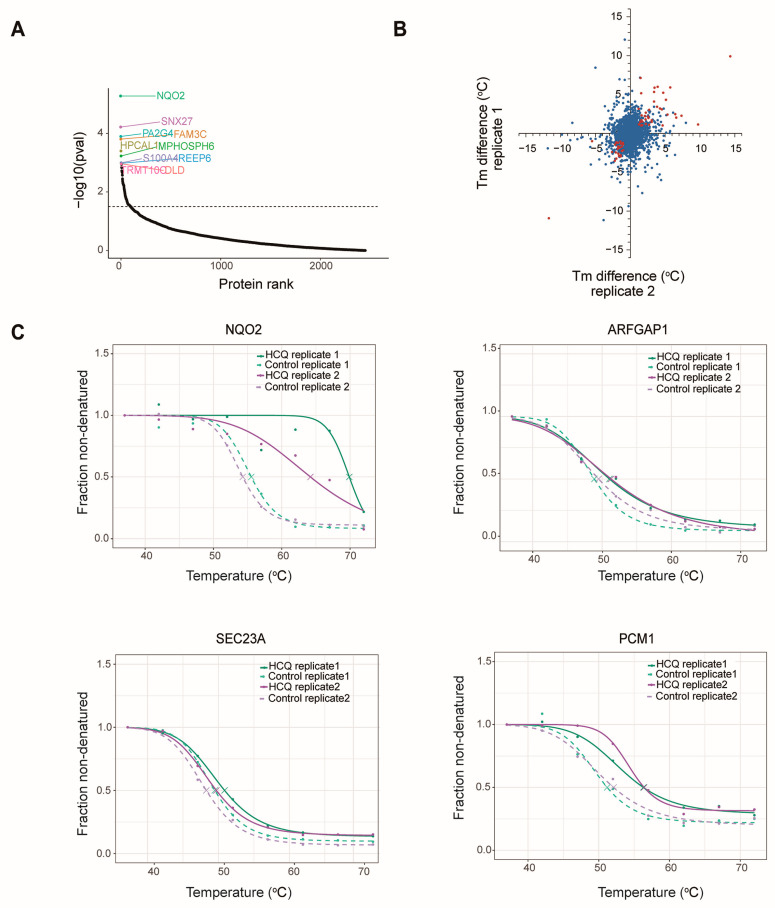
The potential binding proteins of HCQ. (**A**) Ranked proteins according to the *p*-value. A total of 2567 proteins contained sufficient data points for the analyses and 75 of them had *p*-values lower than 0.05. (**B**) Distribution of the proteins according to △Tm in two replicates; blue: total proteins in two replicates; red: proteins with *p*-values lower than 0.05 and |△Tm| higher than 1. (**C**) Thermal melt curves of the representative proteins NQO2, SEC23A, PCM1, and ARFGAP1.

To identify the potential binding proteins of HCQ, we analyzed the thermal proteome profiling data. Firstly, 2932 proteins that could not generate melting curves in either of the two replicates were removed. Through a nonparametric analysis, 75 of the 2567 retained proteins showing significant (*p* < 0.05) HCQ-dependent changes in thermal stability were kept (Figure 7A), including the top-ranked proteins NQO2, sorting nexin-27 (SNX27), and proliferation-associated protein 2G4 (PA2G4). To enhance the reliability, we investigated the intersection of the two biological replicates and picked up 34 proteins exhibiting significant thermal shift (*p* < 0.05 and △Tm > 1) and good repeatability (Figure 7B) (Table S4).

After manually checking, 10 of the 34 proteins were selected as potential binding proteins of HCQ (Table 1, Figure S1). Notably, the thermal stability of three autophagy-related proteins, SEC23A, PCM1, and ARFGAP1, was significantly increased after HCQ treatment (Figure 7C). SEC23A can be phosphorylated by Unc-51-like kinase, leading to the inhibition of substance transport from the endoplasmic reticulum (ER) to the Golgi apparatus and autophagy activation [31]. PCM1 is essential for the correct localization of several centrosomal proteins such as pericentrin (PCNT), serine/threonine-protein kinase Nek2 (NEK2), centrosome-associated protein CEP250 (CEP250), and centrin-3 (CETN3) [32]. It plays an important role in maintaining centrosome organization and stability which is frequently abnormal in cancers [33]. ARFGAP1 is involved in membrane trafficking and can negatively regulate non-canonical autophagy with an interaction of ER-localized protein BPI fold-containing family B member 3 (BPIFB3) [34]. The results suggest that HCQ may inhibit autophagy by targeting these proteins.

Interestingly, the top-ranked protein NQO2 is linked to non-small cell lung cancer [35]. Inhibition of NQO2 can induce the release of reactive oxygen species which can activate ER stress-C/EBP homologous protein and regulate death receptor signaling [35]. Association of the potential binding proteins to diseases suggests that HCQ may play a role in the treatment of certain diseases by targeting these proteins. 

## 3. Discussion

In this study, to explore the mechanisms of action and identify the potential targets of HCQ, we performed a quantitative proteomics study and thermal proteome profiling, respectively. 

In total, 5581 proteins were quantified in the quantitative proteomics study, with 134 and 102 proteins being downregulated and upregulated in response to HCQ treatment, respectively. Among the dynamic proteome induced by HCQ, NBR1, GABARAPL2, LGALS8, and MAPK8 are autophagy-related proteins, suggesting that HCQ may inhibit autophagy by tuning their expression.

Cell pathway analysis showed that the upregulated proteins induced by HCQ are mainly enriched in the cellular metabolism process. As a key node in the metabolic regulating pathways, mTOR controls cellular metabolism and growth by regulating anabolic and catabolic programs, such as protein synthesis and autophagy, respectively [36]. Although the expression of mTOR only has a slight change in response to HCQ treatment, mTOR may be affected by HCQ indirectly. For example, LGALS8, which controls mTOR causing its inactivation and dissociation from damaged lysosomes [21], is downregulated. In addition, tumor necrosis factor alpha-induced protein 8-like protein 1 (TNFAIP8L1), which acts as a negative regulator of mTOR activity [37], is decreased by 60.3% after HCQ treatment. Therefore, HCQ may indirectly activate mTOR by downregulating LGALS8 and TNFAIP8L1 to inhibit autophagy and regulate cellular metabolism.

The global proteomics study has focused on the measurement of protein abundance as a measure of cellular functions of HCQ. This approach, while highly informative, is not sufficient as a standalone approach to identify the targets of HCQ and reveal the roles of HCQ in disease treatments. Thermal proteome profiling is widely used to identify the target proteins of drugs in the principle of thermal shift induced by ligand binding [18]. Therefore, we also performed a thermal proteome profiling study of HCQ in MIA PaCa-2 cells, in which we identified 4144 and 4327 soluble proteins in the HCQ-treated and control groups, respectively. In total, 34 proteins exhibit significantly increased thermal shifts after HCQ treatment. Because HCQ binding may induce the shift in the melting curve of the target protein, these proteins with the significantly increased thermal shift are regarded as potential binding proteins of HCQ. Notably, some of them are involved in autophagy, including SEC23A [38], PCM1 [33], ARFGAP1 [34], and the top-ranked protein NQO2 [35]. Given the roles of NQO2 in non-small cell lung cancer, our results suggest that HCQ may play a role in the treatment of non-small cell lung cancer by targeting NQO2. Furthermore, it also provides some clues to explore the combination treatment of non-small cell lung cancer with HCQ.

It has been demonstrated that HCQ can be used for pancreatic cancer treatment [11]. However, the underlying mechanism remains largely unknown. It was reported that the knockdown of SYNE2 or suppression of ICMT could inhibit the proliferation and invasion of pancreatic cancer [25,26]. Interestingly, our results showed that the expression of protein SYNE2 and ICMT was decreased after HCQ treatment, suggesting that HCQ may inhibit the proliferation of pancreatic cancer cells by reducing the expression of SYNE2 and ICMT. The cell viability assay further confirmed that HCQ can significantly decrease cell proliferation. Another protein of concern was cotranscriptional regulator FAM172A (FAM172A), whose expression was increased by 50% after HCQ treatment. It was reported that the abundance of FAM172A was negatively correlated with pancreatic cancer tumor size and could inhibit epithelial-to-mesenchymal transition (EMT), which eventually led to pancreatic cancer metastasis and highly malignant pancreatic cancer [39]. Therefore, these results provide some clues to the mechanisms by which HCQ exerts its anti-pancreatic cancer effect. 

HCQ is a racemic mixture of two enantiomers and administered as the racemate. Given that enantiomers may exert different pharmacological activities, such as quinine and quinidine [40,41], we could not exclude the possibility that the enantiomers of HCQ may exert different functions in different diseases, although both (*R*) and (*S*) conformations of HCQ have comparable antimalarial activity [42]. 

In conclusion, this study revealed the dynamic proteome induced by HCQ and identified the potential target proteins of HCQ, which not only presents possible mechanisms underlying HCQ-induced autophagy inhibition but may also shed light on the link between HCQ and pancreatic cancer treatment. This analysis provides a valuable starting point to explore the potential targets and action mechanisms of HCQ. However, the targeted proteins of HCQ and more detailed mechanisms in the treatment of pancreatic cancer with HCQ need to be further validated. Once it was experimentally confirmed that HCQ exerts functions through targeting or regulating the proteins revealed in our study, these results may provide clues to explore new indications of HCQ, such as the treatment for non-small cell lung cancer, or develop combination treatments with HCQ for certain diseases, such as pancreatic cancer.

## 4. Materials and Methods

### 4.1. Cell Lines, Antibody, and TMT Agent

The MIA PaCa-2 cell line (catalog number: SCSP-568) was purchased from the National Collection of Authenticated Cell Culture (https://www.cellbank.org.cn, accessed on 21 May 2021) and used without further authentication. The anti-LC3B antibody was purchased from Cell Signaling Technology (catalog number: 2775). The anti-beta actin antibody was purchased from Proteintech (catalog number: 66009-1). The TMT 10plex was purchased from Thermo Fisher (Waltham, MA, USA, catalog number: 90111).

### 4.2. Cell Culture

MIA PaCa-2 cells were seeded in 10 cm dishes (Corning, New York, NY, USA) and cultured at 37 °C in 5% CO_2_ with DMEM plus 10% FBS and 1% penicillin-streptomycin. When the cells grew to about 80%, the experimental and control groups were treated with 250 μM HCQ (purchased from Aladdin, CAS number: 747-36-4) and ddH_2_O for 1.5 h, respectively.

### 4.3. Thermal Proteome Profiling

After incubating with HCQ or ddH_2_O for 1.5 h, MIA PaCa-2 cells were washed with ice-cold PBS three times. Then, the cells were resuspended with ice-cold PBS (containing pepstatin, leupeptin, and PMSF), divided into eight aliquots (96 μL for each), and transferred into 0.2 mL PCR tubes. The tubes were heated in parallel for 3 min at the temperature ranging from 37 °C to 72 °C and followed by cooling for 3 min at 25 °C. After that, 10% NP-40 was added to the tubes at a final concentration of 0.4%, and the tubes were put on ice for 20 min. The tubes were then frozen–thawed three times in liquid nitrogen before ultracentrifugation (16,000× *g* at 4 °C for 10 min). The supernatant was transferred to new tubes and protein concentration was determined by BCA assay. Next, the supernatant was reduced and alkylated with dithiothreitol (5 mM, 56 °C, 30 min) and iodoacetamide (11 mM, room temperature, 30 min), respectively. Finally, the proteins were subjected to short SDS gel electrophoresis.

### 4.4. TMT Labeling

Gel lanes were cut into slices and acetonitrile (ACN) was added. After dehydration, ACN was removed, and the samples were digested by trypsin in 5 mM TEAB buffer (pH 8.0) at 37 °C overnight. Then, the digestion was stopped by adding formic acid to adjust the pH to 2. The peptides were eluted with 50% ACN/1% formic acid (FA) and desalted with a C18-tip. The TMT labeling reaction was performed in 20 μL of 50 mM HEPES at pH 8.5. TMT (50 μg) solution was added to the peptides and the mixture was incubated at 25 °C for 1 h. After the incubation, hydroxylamine was added to a final concentration of 3% to stop the reaction. Subsequently, samples were acidified using 10% FA/10% ACN and evaporated to dryness.

### 4.5. Proteomics Sample Analysis

MIA PaCa-2 cells treated with ddH_2_O or HCQ were lysed using RIPA buffer on ice for 30 min and then sonicated (15W, 3 min) before centrifugation (16,000× *g* at 4 °C for 10 min). After centrifugation, protein concentration was determined by BCA assay and 100 μg of proteins was transferred into new tubes. Next, proteins were precipitated with trichloroacetic acid (TCA) at a final concentration of 10% and washed with pre-cold acetone. Then, the pellet was resuspended with 25 mM Tris buffer (pH 7.5) and digested by trypsin at 37 °C overnight. After digestion, the samples were reduced and alkylated with dithiothreitol (5 mM, 56 °C, 30 min) and iodoacetamide (11 mM, room temperature, 30 min), respectively. 

### 4.6. LC-MS/MS Analysis

For the TPP analysis, 10% FA was added to dissolve peptides and 2 μg of the peptides was loaded on an EASY-nLC 1200 UHPLC system coupled to a Q Exactive HF-X mass spectrometry. Peptides were separated using 10–90% buffer B (80% ACN + 0.1% FA) in 60 min. The mass spectrometer was performed in a data-dependent acquisition mode that can acquire full MS spectra at 60,000 resolution when the automatic gain control (AGC) number is 3 × 10^6^ and the maximum injection time of 50 ms. Then, the top 20 precursors were subjected to be fragmented, with 20 s of dynamic exclusion. The AGC number for MS2 was 5 × 10^4^ and the maximum injection time was 65 ms, acquiring MS2 fragmentation spectra at 45,000 resolution. Moreover, the HCD with an NCE of 28 was used when fragmentation was analyzed.

### 4.7. Data Analysis

The mass data were searched against the UniProt Human protein database (20,376 entries, https://www.uniprot.org, accessed on 9 January 2020) by MaxQuant (v1.6.15.0, Max Planck Institute of Biochemistry, Martinsried, Germany) with quantifying by TMT 10plex labeling. Maximum missed cleavages sites were set as 2. Carbamidomethylation of cysteine was set as the fixed modification. Oxidation of methionine and N-terminal acetylation were set as variable modifications.

To determine melting points, we set the lowest temperature point as 1 and calculated the comparison between the relative abundances of the TMT reporter ions with the lowest temperature. After normalizing the data using the TPP R script (v3.15, European Molecular Biology Laboratory (EMBL), Rome, Italy), we could fit the melting curves of proteins using the following equation.

$$f\left(T\right)=\frac{1-plateau}{1+e^{-\left(\frac{a}{T}-b\right)}}+plateau$$

At this temperature, if half of the protein is denatured, this temperature is the melting point of the protein: f(T)=0.5. 

### 4.8. Bioinformatic Analysis

The cellular pathway analysis based on Gene Ontology (GO) and Reactome pathway database [43] was performed with a hypergeometric test in the clusterProfiler package in R. The protein–protein interaction network of the dynamic proteins induced by HCQ was established based on the STRING database (v11, European Molecular Biology Laboratory (EMBL), Rome, Italy, http://www.string-db.org/, accessed on 19 January 2019) and visualized in Cytoscape (v3.8.2, National Institute of General Medical Sciences (NIGMS), Maryland, MD, USA, https://cytoscape.org/, accessed on 4 July 2022). The functional enrichment of key networks of PPIs was carried out with StringAPP embedded in Cytoscape (v1.7.0, National Institute of General Medical Sciences (NIGMS), Maryland, MD, USA, https://apps.cytoscape.org, accessed on 4 July 2022) [30].

### 4.9. Cell Viability Assay

MIA PaCa-2 cells were seeded in 96-well plates at a density of 1.4 × 10^3^ cells per well in 100 μL of medium. After 24 h, the cells in each well were treated with different concentrations of HCQ for 48 h. Then 10 μL of Cell Counting Kit-8 (CCK-8) regent (Beyotime, catalog number: C0065FT) was added to each well. After incubation for 1 h, the absorbance (A) of each well at 450 nm was measured by a SpectraMax M5e Microplate Reader (Molecular Devices, San Jose, CA, USA).

## Figures and Tables

**Table 1:** The representative potential binding proteins of HCQ.

Gene	△Tm (°C)	Function
NQO2	12	A quinone reductase
GSR	5.8	Maintains high levels of reduced glutathione
NAMPT	2.1	Catalyzes the condensation of nicotinamide
KIF11	2.38	Motor protein required for establishing a bipolar spindle during mitosis
ALDH9A1	1.6	Converts gamma-trimethylaminobutyraldehyde into gamma-butyrobetaine
SEC23A	1.4	Component of the coat protein complex II (COPII)
PUF60	1.3	DNA- and RNA-binding protein
PCM1	4.7	Required for centrosome assembly and function
NONO	1.89	DNA- and RNA binding protein
ARFGAP1	1.98	GTPase-activating protein (GAP) for the ADP ribosylation factor 1 (ARF1)

The table shows the gene names, increased thermal shift, and their function of the representative potential binding proteins of HCQ.

## Data Availability

The mass spectrometry proteomics data have been deposited to the ProteomeXchange Consortium via the PRIDE partner repository with the dataset identifier PXD034196.

## Supplementary Materials

The following supporting information can be downloaded at: https://www.mdpi.com/article/10.3390/molecules27165175/s1. Table S1. The quantitative proteome of MIA PaCa-2 cells in response to HCQ treatment. The data show the dynamics of the proteins induced by HCQ in MIA PaCa-2 cells. Table S2. Biological processes enriched with the differentially regulated proteins by HCQ. Table S3. Reactome pathway enrichment of the differentially regulated proteins by HCQ. Table S4. The proteins exhibiting significantly increased thermal shifts after HCQ treatment. The table shows the potential binding proteins of HCQ identified through the TPP study. Figure S1. Thermal melt curves of proteins poly(U)-binding-splicing factor PUF60 (PUF60), glutathione reductase (GSR), nicotinamide phosphoribosyltransferase (NAMPT), non-POU domain-containing octamer-binding protein (NONO), kinesin-like protein KIF11 (KIF11), and 4-trimethylaminobutyraldehyde dehydrogenase (ALDH9A1).

## Sample Availability

Samples of the HCQ and PSA plus E-64 are available from the authors.
