# Supplementary figures and images for: Quantitative Proteomics Explore the Potential Targets and Action Mechanisms of Hydroxychloroquine
Source: Molecules. 2022 Aug 14;27(16):5175. doi: 10.3390/molecules27165175 (PMC9416215; doi:10.3390/molecules27165175)

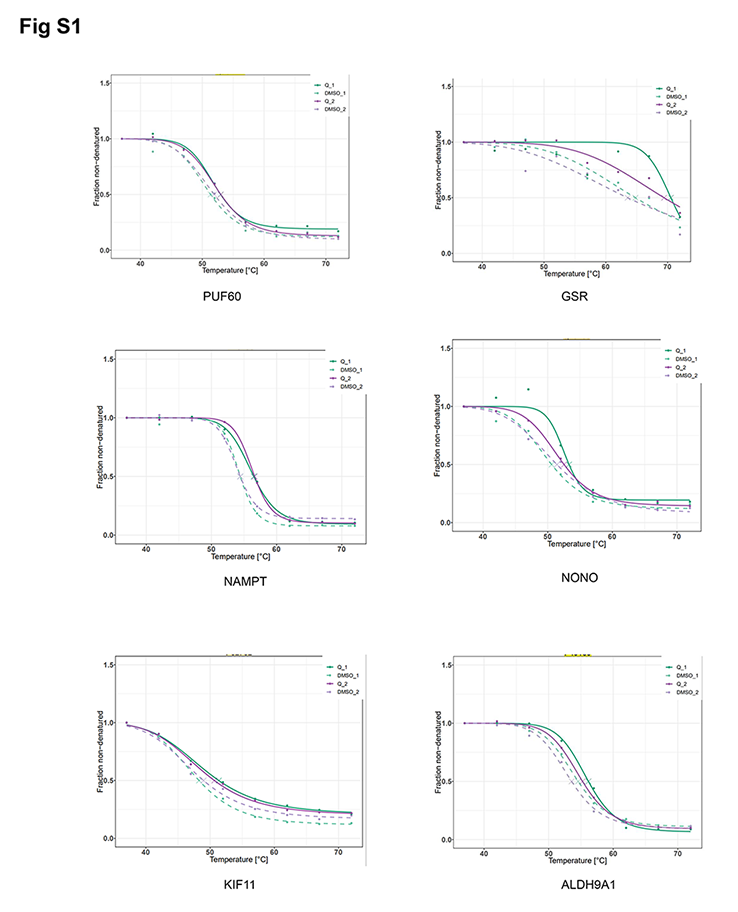

Supplement: Supplementary file 1 [file molecules-27-05175-s001.zip › Figure S1.tif]
